# Embossed topographic depolarisation maps of biological tissues with different morphological structures

**DOI:** 10.1038/s41598-021-83017-2

**Published:** 2021-02-16

**Authors:** Volodimir A. Ushenko, Benjamin T. Hogan, Alexander Dubolazov, Anastasiia V. Grechina, Tatiana V. Boronikhina, Mikhailo Gorsky, Alexander G. Ushenko, Yurii O. Ushenko, Alexander Bykov, Igor Meglinski

**Affiliations:** 1 Chernivtsi National University, 2 Kotsiubynskyi Str., Chernivtsi, 58012 Ukraine; 2 OPEM, ITEE, University of Oulu, 90014 Oulu, Finland; 3 Institute of Clinical Medicine N.V. Sklifosovsky, I.M. Sechenov First Moscow State Medical University, Moscow, 129090 Russia; 4 Bukovinian State Medical University, 3 Theatral Sq., Chernivtsi, 58000 Ukraine; 5 College of Engineering and Physical Science, Aston University, Birmingham, B4 7ET UK

**Keywords:** Imaging and sensing, Biophotonics, Micro-optics, Biomedical engineering, Characterization and analytical techniques, Imaging, 3-D reconstruction, Imaging techniques, Circular dichroism

## Abstract

Layered topographic maps of the depolarisation due to diffuse biological tissues are produced using a polarisation-holographic Mueller matrix method approach. Histological sections of myocardial tissue with a spatially structured optically anisotropic fibrillar network, and parenchymal liver tissue with a polycrystalline island structure are successfully mapped. The topography of the myocardium maps relates to the scattering multiplicity within the volume and the specific morphological structures of the biological crystallite networks. The overall depolarisation map is a convolution of the effects of these two factors. Parenchymal liver tissues behave broadly similarly, but the different biological structures present cause the degree of scattering multiplicity to increase more rapidly with increasing phase. Through statistical analysis, the dependences of the magnitudes of the first to fourth order statistical moments are determined. These moments characterise the changing distributions of the depolarisation values through the volume of biological tissues with different morphological structures. Parenchymal liver tissue depolarisation maps are characterised by larger mean and variance, and less skewness and kurtosis, compared to the distributions for the myocardium. This work demonstrates that a polarisation-holographic Mueller matrix method can be applied to the assessment of the 3D morphology of biological tissues, with applications in disease diagnosis.

## Introduction

Polarimetric diagnostics of optically anisotropic structures in biological tissues are an area of active development in biomedical optics^1–5^. While many techniques have been pursued and investigated, such as scattering matrices^6–8^ and Mueller matrix polarimetry^9–15^, perhaps the most exciting avenue has been Mueller matrix mapping^16–19^. Originally, experimental results obtained by measuring and analysing Mueller matrices were limited to representations as 1D angular dependences of the matrix elements^3,14,20^. However, the advent of digital imaging facilitated an expansion to looking at two-dimensional distributions of the elements of the Mueller matrices^12,13,15^. As such, it became a powerful tool for studying a variety of biological samples, within the framework of approximation models^21–28^. For example, oncological diagnoses could be achieved^19,26,29^. However, such 2D methods consider only the integrally polarised properties, averaged through the full volume cross-section. In contrast, most biological tissues of interest have complex 3D morphologies, formed of linearly and circularly birefringent fibrillar networks^29^. There is hence a clear need for the development of a technique allowing consideration of variance in the third dimension.

Herein we develop and experimentally demonstrate a method for mapping the depolarisation of biological tissues, in three-dimensions, using a Mueller matrix method. The method builds on conventional Stokes polarimetry^1–5,7,8,11^ and interferometry^30,31^ techniques. Algorithms for digital holographic reconstruction^32^ of the amplitude-phase structure of object fields are used to obtain the interrelations between 3D divisions (set of layered distributions) of depolarisation maps. From this, one can then analyse the peculiarities and specific features of polycrystalline structures within histological sections of diffuse biological tissues with different morphologies. The method provides a basis for future biomedical imaging techniques to diagnose adverse conditions.

## Theory and methods

For typical (non-birefringent), diffuse biological tissues (in the volume of which multiple scattering occurs), the magnitude of most elements of the Mueller matrix ${\mathbf{M}}$, is insignificant (or tends to zero)^7,8,12^. However, the elements on the leading diagonal are the clear exceptions. These diagonal matrix elements, ${\mathbf{M}}_{22;33;44}$, determine the degree of depolarisation of light propagating through the optically anisotropic object^1,4,5^. The degree of depolarisation, ${\varvec{\Lambda}}$, can be expressed as:

1 $${\varvec{\Lambda}}=1-\frac{1}{3}\left({\mathbf{M}}_{22}+{\mathbf{M}}_{33}+{\mathbf{M}}_{44}\right)$$

The value of the parameter ${\varvec{\Lambda}}$ is an integral, averaged over the entire volume through which the light propagates, of the optical properties of a biological layer with a thickness $h$. It is determined by the contribution of two mechanisms. Firstly, the formation of an orthogonal component of the amplitude of the laser radiation (i.e. a change to the state of polarisation) due to the optical anisotropy of the biological layer. This is termed the “A component”^1–5^. Secondly, statistical averaging of the state of polarisation due to the superposition of laser waves scattered in the volume of the biological layer with different states of polarisation. This is termed the “B component”^7,8,12^. For an optically homogeneous isotropic layer (${M}_{22;33;44}\to 1$) and hence ${\varvec{\Lambda}}=0$, while for a perfect diffuser (${M}_{22;33;44}\to 0$) and thus ${\varvec{\Lambda}}=1$. In all other cases (i.e. partially depolarizing biological layers), changes in ${\varvec{\Lambda}}$ are determined by the ratio between the A and B components in the longitudinal direction ($z$) within a polycrystalline medium^32^.

**Figure 1:**
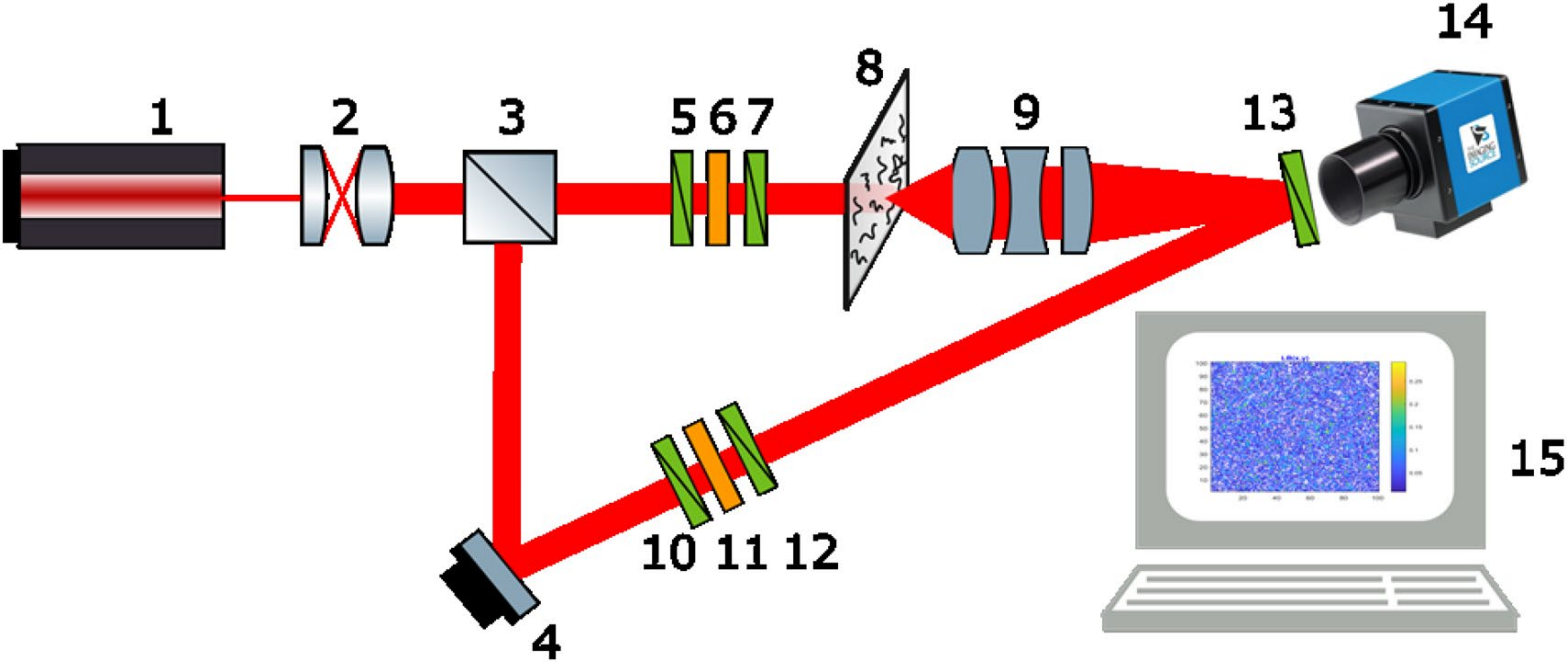
Optical scheme of a 3D Mueller matrix polarimetry setup, consisting of: 1—laser; 2—collimator; 3—beam splitter; 4—reflecting mirror; 5, 7, 10, 12, 13—polarisers; 6,11—quarter-wave plates; 8—object under investigation; 9—strain-free polarisation objective; 14—digital camera; 15—processing unit.

Herein we focus on the possibilities of obtaining information on distributions of the depolarisation parameter ${\varvec{\Lambda}}\left(x,y,{h}_{j}\right)$ within the volume of a biological tissue sample. Figure 1 presents the optical scheme of the modified Stokes polarimeter used for polarisation-holographic Mueller matrix polarimetry measurements of biological layers.

A parallel beam ($\oslash =2\times 1{0}^{3}\upmu \text{m}$) of He–Ne ($\lambda =0.6328 \upmu \text{m}$) laser radiation is generated by passing through the collimator (2). The beam splitter (3) divides the radiation equally into separate illuminating and reference beams. The illuminating beam is given the correct polarisation by rotation of the polarisers (5, 7) and quarter wave plate (6) and directed through the biological layer sample (8). Linear polarisations of 0, 45, 90, and 135° are established by rotating the linear polarisers (5, 7) to the required angle, and by rotating the quarter wave plates (6) so that its fast axis is aligned with the linear polarisation direction such that it has no effect on the polarisation state. Left- and right-circularly polarised states are established by rotating the linear polariser (5) to 45 and 135° respectively relative to the fast axis of the quarter wave plate (6). The inhomogeneous polarisation image of the sample is projected into the plane of a digital camera (14). The reference beam is polarised, as with the illuminating beam, and projected into the plane of the camera. As a result, an interference pattern is formed in the image plane of the camera. The coordinate distribution of the intensity of the interference pattern is recorded.

The polarisation-holographic determination of the Mueller matrix elements follows the following procedure. First, six distinct polarisation state sets are formed in the illuminating and reference laser beams—($\left({0}^{0}-{0}^{0}\right);$ $\left({45}^{0}-{45}^{0}\right);$ $\left(9{0}^{0}-9{0}^{0}\right);$ $\left({135}^{0}-{135}^{0}\right);$ $\left(\otimes -\otimes \right);$ and $\left(\oplus -\oplus \right)$). Here, $\otimes {\mathrm{ and }} \oplus$ designate right and left circular polarisations respectively. For each polarisation state set, the camera records the partial interference image formed by superimposing the reference wave on the polarisation-filtered image of the sample. Polarisation filtering is carried out by means of a polarizer-analyser with sequential orientation of its transmission plane at the angles $$\begin{array}{cc}{\Theta }_{x}={0}^{0};& {\Theta }_{y}=9{0}^{0}\end{array}$$.

A direct fast Fourier transform of the partial interference images is performed and a complex amplitude image of the spatial frequencies is determined in the form of two components with a frequency shifted by the frequency of the interference pattern. One of these components is cut off and the inverse Fourier transform is performed, providing a reconstruction of the complex wavefront of the biological tissue sample. The distribution of complex amplitudes is given by $$\left\{\begin{array}{l}{\Theta }_{\mathrm{x}}\to \left|{\mathrm{U}}_{\mathrm{x}}\right|;\\ {\Theta }_{\mathrm{y}}\to \left|{\mathrm{U}}_{\mathrm{y}}\right|{\text{e}}{\text{x}}{\text{p}}\left({\mathrm{i}}\left({\upphi }_{\mathrm{y}}-{\upphi }_{\mathrm{x}}\right)\right)\end{array}\right.$$. Backpropagation of the complex wavefront is evaluated to determine the wavefront at specific depth positions within the sample. The position of these planes along the $z$ axis is determined by the phase $$\begin{array}{cc}{\uptheta }_{\mathrm{j}}={\left({\upphi }_{\mathrm{y}}-{\upphi }_{\mathrm{x}}\right)}_{\mathrm{j}}=\frac{2 \pi}{\uplambda }\text{z;}& 0\le {\mathrm{z}}\le {\mathrm{h}}\end{array}$$, of the object field. The planes are separated by an arbitrary step of ${\Delta}{\theta}_{{\mathrm{j}}=0 \ldots {\text p}}$.

For each state of the illuminating beam, the reconstructed distributions of the Stokes vector parameters ($SV$) in the set of phase planes ${\theta }_{k}$ are calculated from the distributions of complex amplitudes $$\left\{\begin{array}{cc}{U}_{x}\left(x,y\right) ;& {U}_{y}\left(x,y\right)\end{array}\right\}\left({\theta }_{k}\right)$$:

2 $$SV{\left({U}_{x},{U}_{y},{\theta }_{k}\right)}^{\left({0}^{0},9{0}^{0},4{5}^{0},\otimes \right)}=\left(\begin{array}{c}{\left({\left|{U}_{x}\right|}^{2}+{\left|{U}_{y}\right|}^{2}\right)}^{\left({0}^{0},9{0}^{0},4{5}^{0},\otimes \right)};\\ {\left({\left|{U}_{x}\right|}^{2}-{\left|{U}_{y}\right|}^{2}\right)}^{\left({0}^{0},9{0}^{0},4{5}^{0},\otimes \right)};\\ 2{\mathit{Re}}\left|{U}_{x}\right|{\left|{U}_{y}\right|}^{\left({0}^{0},9{0}^{0},4{5}^{0},\otimes \right)};\\ 2{\mathit{Im}}\left|{U}_{x}\right|{\left|{U}_{y}\right|\left({0}^{0},9{0}^{0},4{5}^{0},\otimes \right)}_{.}\end{array}\right)({\theta }_{k})$$

The set of layered distributions of Mueller matrix elements ${M}_{ik}(x,y,{\theta }_{k})$ is calculated by the following Stokes-polarimetric relations. For the Stokes vectors of linearly polarised probing beams $S{{V}_{0}}^{\left({0}^{0}\right)}$ and $S{{V}_{0}}^{\left(9{0}^{0}\right)}$:

3 $$\left\{\begin{array}{c}\left[S{{V}_{0}}^{\left({0}^{0}\right)}=\left\{M\right\}\left(\begin{array}{c}1\\ 1\\ 0\\ 0\end{array}\right)\to S{V}^{\left({0}^{0}\right)}\left({U}_{x},{U}_{y},{\theta }_{k}\right)=\left(\begin{array}{c}{M}_{11}+{M}_{12}\\ {M}_{21}+{M}_{22}\\ {M}_{31}+{M}_{32}\\ {M}_{41}+{M}_{42}\end{array}\right)\right];\\ \left[S{{V}_{0}}^{\left(9{0}^{0}\right)}=\left\{M\right\}\left(\begin{array}{c}1\\ -1\\ 0\\ 0\end{array}\right)\to S{V}^{\left(9{0}^{0}\right)}\left({U}_{x},{U}_{y},{\theta }_{k}\right)=\left(\begin{array}{c}{M}_{11}-{M}_{12}\\ {M}_{21}-{M}_{22}\\ {M}_{31}-{M}_{32}\\ {M}_{41}-{M}_{42}\end{array}\right)\right]\end{array}\right\}\Rightarrow {M}_{ik}= \left \Vert \begin{array}{cc}{M}_{11}& {M}_{12}\\ {M}_{21}& {M}_{22}\\ {M}_{31}& {M}_{32}\\ {M}_{41}& {M}_{42}\end{array} \right \Vert \left({U}_{x},{U}_{y},{\theta }_{k}\right)$$

For the Stokes vectors of linearly polarised probing beams $$S{V}_{0}\begin{array}{cc}\left(4{5}^{0}\right);& S{{V}_{0}}^{\left(13{5}^{0}\right)}\end{array}$$:

4 $$\left\{\begin{array}{c}\left[S{{V}_{0}}^{\left(4{5}^{0}\right)}=\left\{M\right\}\left(\begin{array}{c}1\\ 0\\ 1\\ 0\end{array}\right)\to S{V}^{\left(4{5}^{0}\right)}\left({U}_{x},{U}_{y},{\theta }_{k}\right)=\left(\begin{array}{c}{M}_{11}+{M}_{13}\\ {M}_{21}+{M}_{23}\\ {M}_{31}+{M}_{33}\\ {M}_{41}+{M}_{43}\end{array}\right)\right];\\ \left[S{{V}_{0}}^{\left(13{5}^{0}\right)}=\left\{M\right\}\left(\begin{array}{c}1\\ 0\\ -1\\ 0\end{array}\right)\to S{V}^{\left(13{5}^{0}\right)}\left({U}_{x},{U}_{y},{\theta }_{k}\right)=\left(\begin{array}{c}{M}_{11}-{M}_{13}\\ {M}_{21}-{M}_{23}\\ {M}_{31}-{M}_{33}\\ {M}_{41}-{M}_{43}\end{array}\right)\right]\end{array}\right\}\Rightarrow {M}_{ik}= \left \Vert \begin{array}{cc}{M}_{11}& {M}_{13}\\ {M}_{21}& {M}_{23}\\ {M}_{31}& {M}_{33}\\ {M}_{41}& {M}_{43}\end{array} \right \Vert \left({U}_{x},{U}_{y},{\theta }_{k}\right)$$

And lastly, for the Stokes vectors of right- and left-circularly polarized probing beams $$S{V}_{0}\begin{array}{cc}\left(\otimes \right);& S{{V}_{0}}^{\left(\oplus \right)}\end{array}$$:

5 $$\left\{\begin{array}{c}\left[S{{V}_{0}}^{\left(\otimes \right)}=\left\{M\right\}\left(\begin{array}{c}1\\ 0\\ 0\\ 1\end{array}\right)\to S{V}^{\left(\otimes \right)}\left({U}_{x},{U}_{y},{\theta }_{k}\right)=\left(\begin{array}{c}{M}_{11}+{M}_{14}\\ {M}_{21}+{M}_{24}\\ {M}_{31}+{M}_{34}\\ {M}_{41}+{M}_{44}\end{array}\right)\right];\\ \left[S{{V}_{0}}^{\left(\oplus \right)}=\left\{M\right\}\left(\begin{array}{c}1\\ 0\\ 0\\ -1\end{array}\right)\to S{V}^{\left(\oplus \right)}\left({U}_{x},{U}_{y},{\theta }_{k}\right)=\left(\begin{array}{c}{M}_{11}-{M}_{14}\\ {M}_{21}-{M}_{24}\\ {M}_{31}-{M}_{34}\\ {M}_{41}-{M}_{44}\end{array}\right)\right]\end{array}\right\}\Rightarrow {M}_{ik}= \left \Vert \begin{array}{cc}{M}_{11}& {M}_{14}\\ {M}_{21}& {M}_{24}\\ {M}_{31}& {M}_{34}\\ {M}_{41}& {M}_{44}\end{array} \right \Vert \left({U}_{x},{U}_{y},{\theta }_{k}\right)$$

We then obtain working relations for determining the values of the Mueller matrix elements:

6 $$\left\{M\right\}\left({U}_{x},{U}_{y},{\theta }_{k}\right)={\mathrm{0,5}} \left \Vert \begin{array}{cccc}\left(S{V}_{1}^{\left({0}^{0}\right)}+S{V}_{1}^{\left(9{0}^{0}\right)}\right)& \left(S{V}_{1}^{\left({0}^{0}\right)}-S{V}_{1}^{\left(9{0}^{0}\right)}\right)& \left(S{V}_{1}^{\left(4{5}^{0}\right)}-S{V}_{1}^{\left(13{5}^{0}\right)}\right)& \left(S{V}_{1}^{\left(\otimes \right)}-S{V}_{1}^{\left(\oplus \right)}\right)\\ \left(S{V}_{2}^{\left({0}^{0}\right)}+S{V}_{2}^{\left(9{0}^{0}\right)}\right)& \left(S{V}_{2}^{\left({0}^{0}\right)}-S{V}_{2}^{\left(9{0}^{0}\right)}\right)& \left(S{V}_{2}^{\left(4{5}^{0}\right)}-S{V}_{2}^{\left(13{5}^{0}\right)}\right)& \left(S{V}_{2}^{\left(\otimes \right)}-S{V}_{2}^{\left(\oplus \right)}\right)\\ \left(S{V}_{3}^{\left({0}^{0}\right)}+{S}_{3}^{\left(9{0}^{0}\right)}\right)& \left(S{V}_{3}^{\left({0}^{0}\right)}-S{V}_{3}^{\left(9{0}^{0}\right)}\right)& \left(S{V}_{3}^{\left(4{5}^{0}\right)}-S{V}_{3}^{\left(13{5}^{0}\right)}\right)& \left(S{V}_{3}^{\left(\otimes \right)}-S{V}_{3}^{\left(\oplus \right)}\right)\\ \left(S{V}_{4}^{\left({0}^{0}\right)}+S{V}_{4}^{\left(9{0}^{0}\right)}\right)& \left(S{V}_{4}^{\left({0}^{0}\right)}-S{V}_{4}^{\left(9{0}^{0}\right)}\right)& \left(S{V}_{4}^{\left(4{5}^{0}\right)}-S{V}_{4}^{\left(13{5}^{0}\right)}\right)& \left(S{V}_{4}^{\left(\otimes \right)}-S{V}_{4}^{\left(\oplus \right)}\right)\end{array} \right \Vert$$

Therefore, the polarisation-holographic Mueller matrix mapping results in the set of layered (${\theta }_{k}$) two-dimensional ($x,y$) distributions of the values of depolarisation degree (${\varvec{\Lambda}}$):

7 $${\varvec{\Lambda}}\left(x,y,{\theta }_{k}\right)=1-\frac{1}{3}\left\{\left[\left(S{V}_{2}^{\left({0}^{0}\right)}-S{V}_{2}^{\left(9{0}^{0}\right)}\right)\right]+\left[\left(S{V}_{3}^{\left(4{5}^{0}\right)}-S{V}_{3}^{\left(13{5}^{0}\right)}\right)\right]+\left[\left(S{V}_{4}^{\left(\otimes \right)}-S{V}_{4}^{\left(\oplus \right)}\right)\right]\right\}$$

The distributions of the values $\Lambda \left(\text{x,y,}{\theta}_{\mathrm{k}}\right)$ can then be quantitatively assessed by calculating the aggregate central statistical moments of the first to fourth orders ${\mathrm{Z}}_{{\mathrm{i}}=\text{1;2;3;4}}$^14^ in each phase plane ${\uptheta }_{\mathrm{k}}$. The first order moment, ${Z}_{1}$, is the mean distribution of the magnitude of the degree of depolarisation (integral value) ${\varvec{\Lambda}}$; ${Z}_{2}$ is the variance of the distribution of the magnitude of the degree of depolarisation ${\varvec{\Lambda}}$; ${Z}_{3}$ is the skewness coefficient of the distribution of the magnitude of the degree of depolarisation ${\varvec{\Lambda}}$; and ${Z}_{4}$ is the kurtosis coefficient of the distribution of the magnitude of the degree of depolarisation ${\varvec{\Lambda}}$.

## Results and discusssion

To demonstrate the method, histological sections of biological tissues with different morphological structures were investigated. Samples were obtained by the standard method on a frozen microtome. This study was conducted in accordance with the principles of the Declaration of Helsinki, and in compliance with the International Conference on Harmonization-Good Clinical Practice and local regulatory requirements. Ethical approval was obtained from the Ethics Committee of the Bureau of Forensic Medicine of the Chernivtsi National University and the Bukovinian State Medical University (Chernivtsi, Ukraine), and written informed consent was obtained from all subjects prior to study initiation.

Two types of tissues with different morphological and optically anisotropic structures were considered:

1. Depolarising ($\Lambda =85\%$), optically thick (attenuation coefficient $\uptau =2.08$), layers of myocardial fibrillar tissue. These tissues have a spatially structured optically anisotropic mesh of protein fibrils, which are formed by optically active myosin molecules. The anisotropic mesh gives rise to linear birefringence and dichroism. The myosin molecules are responsible for circular birefringence and dichroism^13–15,26^.
2. Parenchymal liver tissue ($\uptau =2.04;\Lambda =82 \%$). The optically anisotropic components of such tissues are small-scale "island" structures formed by polypeptide chains of optically active protein molecules^13–15,26^.

**Figure 2:**
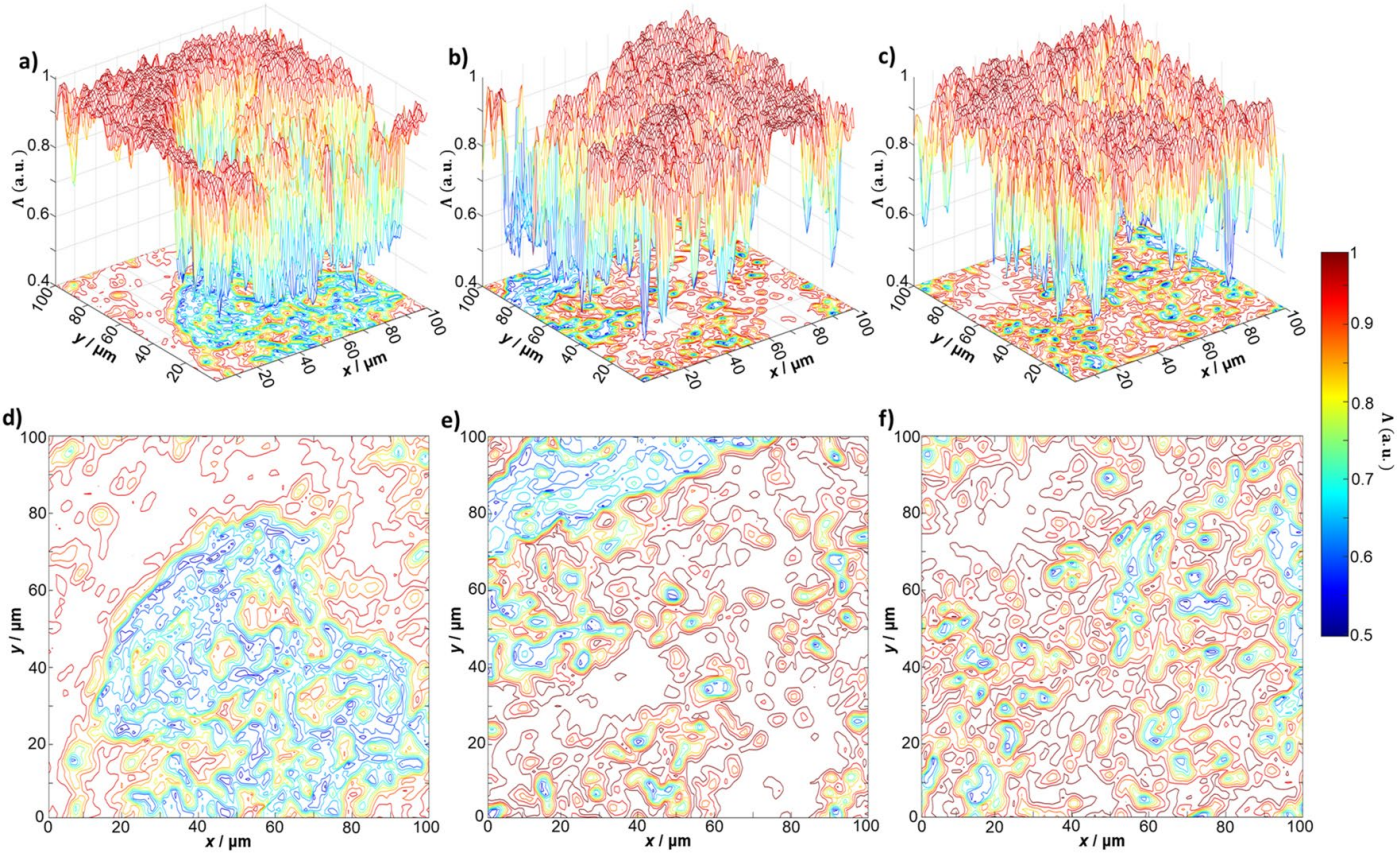
The layered distribution of the degree of depolarisation of the histological section of the myocardium. The distribution is shown as (**a**–**c**) 3D surfaces and (**d–f**) 2D contours, for the phase sections at (**a**,** d**) ${\theta }_{k}=\text{0.3 rad}$, (**b**, **e**) ${\theta }_{k}=\text{0.9 rad}$, and (**c**, **f**) ${\theta }_{k}=\text{1.5 rad}$ respectively. Data visualised using the meshc and contour plot functions in Matlab R2020a (www.mathworks.com).

**Figure 3:**
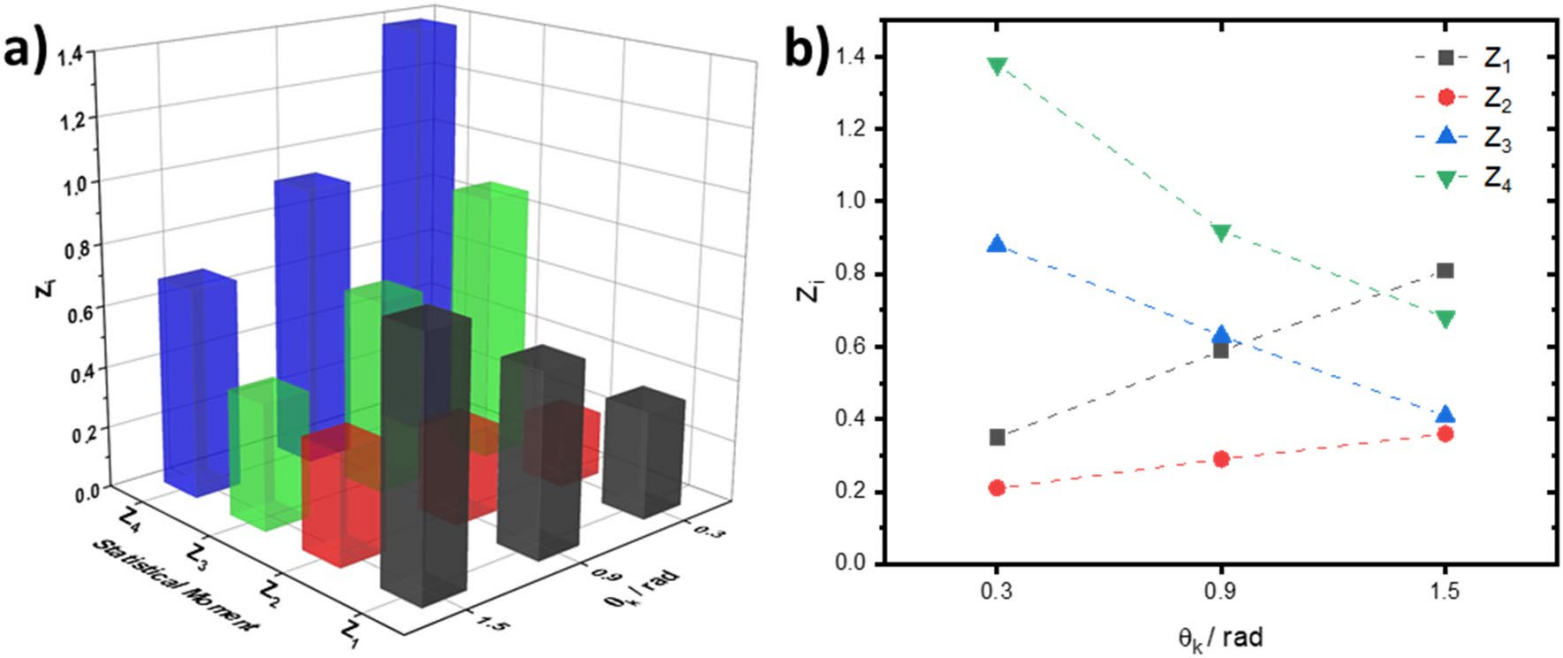
The statistical moments of the first to fourth orders, as calculated in three phase sections of a embossed topographic depolarisation map of a myocardial tissue sample. Data was plotted in OriginPro 2020 (www.originlab.com).

Figure 2 shows a series of layered depolarisation maps $\Lambda \left(\text{x,y}\right)$ of the histological section of the myocardium for phase sections at: ${\uptheta }_{\mathrm{k}}=\text{0.3 rad}$ (see Fig. 2a,d); ${\uptheta }_{\mathrm{k}}=\text{0.9 rad}$, (see Fig. 2b,e); and ${\uptheta }_{\mathrm{k}}=\text{1.5 rad}$, (see Fig. 2c,f). Figure 3 gives the values of the first to fourth order (statistical moments mean, variance, skewness, and kurtosis) characterising the distribution of the degree of depolarisation in each phase plane. One can see immediately that the depolarisation in each phase section ${\uptheta }_{\mathrm{k}}$ is characterised by a different statistical structure, such that ${\mathrm{Z}}_{{\mathrm{i}}=\text{1;2;3;4}}\left({\uptheta }_{\mathrm{j}}\right)\ne {\mathrm{Z}}_{{\mathrm{i}}=\text{1;2;3;4}}\left({\uptheta }_{{\mathrm{j}}+1}\right)$. An increase in the mean ${\mathrm{Z}}_{1}\left(\uptheta \right)$ and variance ${\mathrm{Z}}_{2}\left(\uptheta \right)$, characterising the distribution, is observed with increasing ${\uptheta }_{\mathrm{k}}$. A decrease in the magnitude of the higher order statistical moments (${\mathrm{Z}}_{3}\left(\uptheta \right)$ and ${\mathrm{Z}}_{4}\left(\uptheta \right)$), characterising the skewness and kurtosis of the distributions occurs for increasing ${\uptheta }_{\mathrm{k}}$.

The topographic structure of the myocardium phase section maps (see Fig. 2d–f) can be related to different ratios between the A and B components of the degree of depolarisation ${\varvec{\Lambda}}$ in different phase planes ${\uptheta }_{\mathrm{k}}$. In the low-multiplicity scattering region (${\uptheta }_{\mathrm{k}}=\text{0.3 rad}$), the A component prevails. With a longitudinal increasing of ${\uptheta }_{\mathrm{k}}$ (an increase in the light scattering multiplicity), the contribution of the B component increases. As a result we have an increase in the value of $\boldsymbol{\Lambda }\left(\text{x,y,}{\theta}_{\mathrm{k}}\right)$ for the same (x,y) position. According to the central limit theorem^33^, there is then a tendency to form normally distributed values of ${\varvec{\Lambda}}$. That is, if the mean, ${\mathrm{Z}}_{1}\left(\Lambda \right)$, and variance ${\mathrm{Z}}_{2}\left(\Lambda \right)$ increase, then conversely the skewness ${\mathrm{Z}}_{3}\left(\Lambda \right)$ and kurtosis ${\mathrm{Z}}_{4}\left(\Lambda \right)$ decrease, as we observe in Fig. 3.

**Figure 4:**
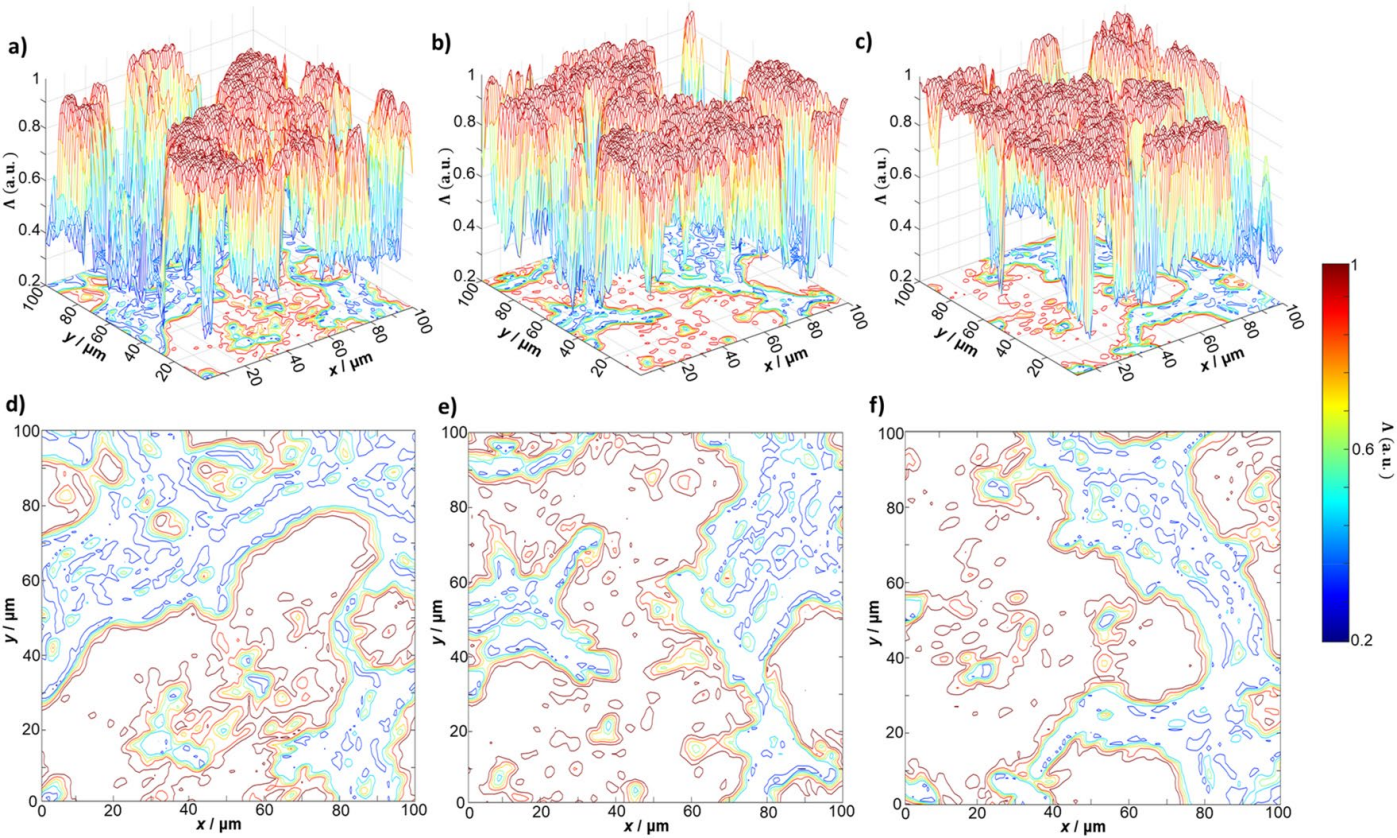
The layered distribution of the degree of depolarisation of a histological section of parenchymal liver tissue. The distribution is shown as (**a**–**c**) 3D surfaces and (**d**–**f**) 2D contours, for the phase sections at (**a**, **d**) ${\theta }_{k}=\text{0.3 rad}$, (**b**, **e**) ${\theta }_{k}=\text{0.9 rad}$, and (**c**, **f**) ${\theta }_{k}=\text{1.5 rad}$ respectively. Data visualised using the meshc and contour plot functions in Matlab R2020a (www.mathworks.com).

**Figure 5:**
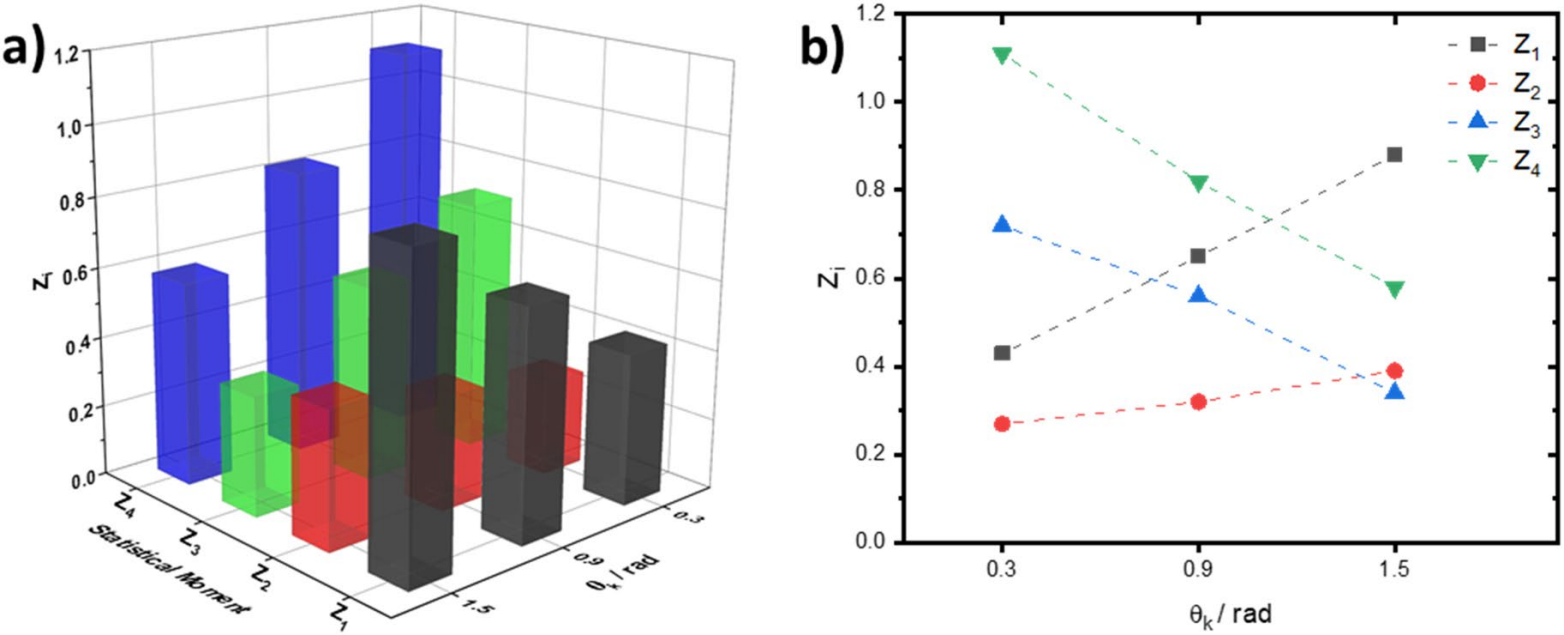
The statistical moments of the first to fourth orders, as calculated in three phase sections of a 3D depolarisation map of a liver tissue sample. Data was plotted in OriginPro 2020 (www.originlab.com).

Comparing the layered depolarisation maps of the myocardium tissue (see Fig. 2) with those of parenchymal liver tissue (Fig. 4), one observes a slightly higher average (${Z}_{1}\left({\theta }_{k}\right)$) level of depolarisation in the set of phase planes ${\theta }_{k}$ of parenchymal liver tissue. Figure 5 gives the values of the statistical moments characterising the distribution of the degree of depolarisation in different phase planes in the parenchymal liver tissue. Analysis of the statistical structure of the depolarisation maps of liver tissue demonstrates broad similarity with the results obtained for fibrillar myocardial tissue (see Fig. 3). However, there are also differences. The optically anisotropic component of the sample of the parenchymal liver tissue is small-scale "island" structures, formed by birefringent polypeptide chains of optically active protein molecules (affecting the A component). Hence, the diffraction angle of the laser radiation is much larger than the analogous parameter for large-scale fibrillar networks. In the limit of small ${\theta }_{k}$ the contributions of the different A and B components to the formation of the magnitude of the degree of depolarisation are comparable. Therefore, the distributions for small values of the phase section (${\uptheta }_{\mathrm{k}}<\text{0.6 rad}$) are characterised by large mean and variance, and conversely, less skewness and kurtosis compared to the distributions for the myocardium.

The value of the integral (within the entire volume of the biological tissue layer) degree of depolarisation ${\varvec{\Lambda}}$ is determined by the influence of the B component. This component depends on both the attenuation (extinction) $\tau$ and geometric thickness $h$. Therefore, the greater the attenuation and geometric thickness of a layer of any tissue, the higher the level of the traditionally measured integral depolarisation. In our case, tissue samples with different morphological structure (fibrillar myocardium and parenchymal liver) are characterized by comparable parameters $\tau$ and $h$. Therefore, against the background of diffuse scattering, differences in the optically anisotropic structure of these samples practically do not appear, and the integral level of depolarisation turns out to be comparable. A different situation takes place in the “intermediate” phase planes (${\theta }_{k}=\text{0.3 rad}$ and ${\theta }_{k}=\text{0.9 rad}$). Here, the influence of the B-component is higher for liver samples due to the greater diffraction expansion and the subsequent superposition of differently polarized partial wave fronts, which are formed by the A-component. With increasing ${\theta }_{k}$, the contribution of the B component becomes decisive for samples of both types. Therefore, the differences between the central statistical moments ${Z}_{i=\text{1;2;3;4}}\left({\theta }_{k}=\text{1.5rad}\right)$ that characterize the distribution of the magnitude of the degree of depolarisation $\Lambda \left({\theta }_{k}=\text{1.5rad}\right)$ are minimized for both types of biological tissues.

To check the reproducibility of the results and determine the reliability of the method, statistical measurements were carried out for two representative samplings of histological sections of the myocardium ($n=36$ samples) and liver ($n=36$ samples). To determine the statistical reliability of the method, the root-mean-square deviation ${\sigma }^{2}$ of the measured values ${Z}_{i={\mathrm{1,2}},\mathrm{3,4}}(n)$ characterising the distribution of the integral degree of depolarisation ${\varvec{\Lambda}}$ was determined. The specified number of samples provided the level of ${\sigma }^{2}\le 0.025$. This deviation corresponds to the value of the confidence interval $p<0.05$. Table 1 shows the mean values of the central statistical moments ${\bar{Z}}_{i=\text{1;2;3;4}}$ and their mean errors $\pm 2\sigma$, which characterise the layered distributions $\Lambda \left(\text{x,y,}{\theta}_{\mathrm{k}}\right)$.

**Table 1:** Mean statistical moments of sets of layered depolarisation maps of histological sections of the myocardium and liver.

Tissue type	Myocardium ($n={36}$)				Liver ($n={36}$)			
${Z}_{i=1;2;3;4}$	${\bar{Z}}_{1}\pm 2\sigma$	${\bar{Z}}_{2}\pm 2\sigma$	${\bar{Z}}_{3}\pm 2\sigma$	${\bar{Z}}_{4}\pm 2\sigma$	${\bar{Z}}_{1}\pm 2\sigma$	${\bar{Z}}_{2}\pm 2\sigma$	${\bar{Z}}_{3}\pm 2\sigma$	${\bar{Z}}_{4}\pm 2\sigma$
${\theta }_{k}=\text{0.3 rad}$	0.37 ± 0.016	0.22 ± 0.009	0.86 ± 0.041	1.33 ± 0.063	0.41 ± 0.018	0.27 ± 0.012	0.77 ± 0.036	1.18 ± 0.054
${\theta }_{k}=\text{0.9 rad}$	0.57 ± 0.024	0.27 ± 0.012	0.63 ± 0.029	0.99 ± 0.044	0.65 ± 0.031	0.33 ± 0.014	0.51 ± 0.023	0.77 ± 0.036
${\theta }_{k}=\text{1.5 rad}$	0.81 ± 0.038	0.35 ± 0.016	0.39 ± 0.017	0.61 ± 0.027	0.84 ± 0.039	0.37 ± 0.017	0.33 ± 0.014	0.53 ± 0.023

The analysis of the obtained statistical data (Table 1) confirms the regularities of the formation of layered depolarisation maps ${\varvec{\Lambda}}\left({\theta}_{\mathrm{k}}\right)$. For optically thick (diffuse) histological sections of the spatially structured myocardium and parenchymal liver with an increase ($\uparrow$) in the value of $\theta {}_{k}$: ${Z}_{i=\text{1;2}}\uparrow$ and ${Z}_{i=\text{3;4}}\downarrow$. The most sensitive to changes in the mechanisms of formation of depolarisation by optically anisotropic (A component) and diffuse (B component) samples of the studied biological tissues were skewness ${Z}_{3}\left({\theta }_{k}\right)$ and kurtosis ${Z}_{4}\left({\theta }_{k}\right)$. The maximum differences between them were found for the phase section: ${\theta }_{k}=\text{0.9 rad}$ where $\Delta {\bar{Z}}_{3}=0.12$ and $\Delta {\bar{Z}}_{4}=0.22$.

## Conclusion

The polarisation-holographic Mueller matrix method of layered mapping of depolarisation maps of diffuse layers of biological tissues with different morphological structures is proposed, described and demonstrated. Changes in the statistical moments characterising the distribution of the degree of depolarisation of optically thick layers of the myocardium ($\uptau =2.08;\Lambda =87\%$) and liver ($\uptau =2.14;\Lambda =88\%$) in different phase sections of their volume were investigated and analysed. The topographic depolarisation structure of the myocardium phase section maps can be related to two main factors—the scattering multiplicity within the volume, and the specific morphological structures of the biological crystallite networks. The overall depolarisation map is a convolution of the effects of these two factors. Liver tissues behave broadly similarly, although there are some key differences. The different biological structures present cause the degree of scattering multiplicity to increase more rapidly for increasing phase. Analysis of the statistical moments of the depolarisation maps demonstrates that the distributions are characterised by larger mean and variance, and less skewness and kurtosis, compared to the distributions for the myocardium.
